# Thiourea-Isocyanate-Based Covalent Organic Frameworks with Tunable Surface Charge and Surface Area for Methylene Blue and Methyl Orange Removal from Aqueous Media

**DOI:** 10.3390/mi13060938

**Published:** 2022-06-13

**Authors:** Selin S. Suner, Sahin Demirci, Duygu S. Sutekin, Selehattin Yilmaz, Nurettin Sahiner

**Affiliations:** 1 Department of Chemistry & Nanoscience and Technology Research and Application Center, Canakkale Onsekiz Mart University Terzioglu Campus, Canakkale 17100, Turkey; sagbasselin@gmail.com (S.S.S.); sahindemirci@gmail.com (S.D.); seletyilmaz@hotmail.com (S.Y.); 2 Department of Chemistry, Hacettepe University, Beytepe, Ankara 06800, Turkey; duygu@hacettepe.edu.tr; 3 Department of Chemical and Biomolecular Engineering, University of South Florida, Tampa, FL 33620, USA; 4 Department of Ophthalmology, Morsani College of Medicine, University of South Florida, 12901 Bruce B Downs B. Downs Blv., MDC 21, Tampa, FL 33612, USA

**Keywords:** porous coordination polymers, covalent organic framework, thiourea-isocyanate, absorbent, dye removal

## Abstract

A thiourea hexamethylene diisocyanate covalent organic framework (TH COF) was synthesized by adjusting the surface charge and surface area. The surface charge value of TH COF, −3.8 ± 0.5 mV, can be changed to −29.1 ± 0.4 mV by treatment with NaOH (dp-TH) and 17.1 ± 1.0 mV by treatment with HCl (p-TH). Additionally, the surface area of TH COF was 39.3 m^2^/g, whereas the surface area of dp-TH COF and p-TH COF structures were measured as 41.4 m^2^/g and 42.5 m^2^/g, respectively. However, the COF structure had a better adsorption capability with acid and base treatments, e.g., dp-TH COF absorbed 5.5 ± 0.3 mg/g methylene blue (MB) dye, and p-TH COF absorbed 25.9 ± 1.4 mg/g methyl orange (MO) dye from 100 mL 25 ppm aqueous dye solutions, thereby increasing the MB and MO absorption amounts of the TH COF structure. Furthermore, by calculating the distribution, selectivity, and relative selectivity coefficients, the absorption capacity order was determined as dp-TH > TH > p-TH COFs for the MB dye, whereas it was p-TH > TH > dp-TH COFs for the MO dye. Finally, the reusability of dp-TH COF for MB absorption and p-TH COF for MO absorption were investigated. After five repeated uses, dp-TH COF retained 64.6 ± 3.7% of its absorption ability, whereas p-TH COF preserved 79.7 ± 3.2% of its absorption ability relative to the initial absorption amount.

## 1. Introduction

The World Health Organization stated that only 2.5% of the water on Earth is clean or potable water and 70% of it is in a solid-state in glaciers [1]. In addition, according to a report of the United Nations World Water Development, more than 2 million tons of water per day is transformed into wastewater for industrial, chemical, agricultural, etc. reasons [2]. More than 100,000 different types of dyes are used in paper, textile, leather, dye, plastic, cosmetic industries, and pharmaceutical applications. As a result of the increasing industrial use of dyes, the removal of these pollutants from water becomes a significant problem due to their toxic, teratogenic, and mutagenic hazards to human health and their harmful effects on the ecosystem [3,4,5]. Due to insufficient and gradually decreasing water resources, studies about removing toxic substances from water resources and the reuse of water have gained enormous importance. Since most of the dyestuffs are stable in relation to light, oxidation agents, and aerobic digestion, the self-destruction of dyes in nature requires a long and difficult process [6,7]. Therefore, many methods were developed to remove dyes, such as membrane filtration, coagulation–flocculation, adsorption, aerobic–anaerobic application, electrochemical techniques, etc. [6,8]. However, the adsorption/absorption method has evolved into one of the most effective physical processes for the removal of dyestuff [9,10,11,12,13]. The most important features of an ideal adsorbent/absorbent material are durability, porosity, multifunctionality, and a large surface area [14].

Covalent organic frameworks (COFs) are crystalline porous oligomers/polymers that form strong covalent bonds with structural units and can be described as a new superior version of organic zeolites. COFs were first discovered and reported by Yaghi and colleagues in 2005 [15] and many developments in the application of COFs have been rapidly addressed by many interdisciplinary researchers working in the fields of organic and polymer chemistry and materials science. Thanks to the superior physical and chemical properties of COF materials such as a large surface area, an adjustable pore size, a high crystallinity, designability, and various molecular architectures [16,17], they are used in many special applications such as gas storage [18], catalysis [19,20], optoelectric [21], electrochemical energy storage [22], sensor [23], drug distribution [24], the capture of water pollutants [25,26], etc. [27,28,29]. The success of porous materials in the industry is linked to their mechanical resistance against impacts such as pulling, pressing, cutting, bending, or twisting [30]. Porous COF structures have excellent hydrolytic stability and are also resistant to degradation by hydrolysis against water, i.e., they do not degrade or undergo structural changes in water [31]. In this study, covalently bound covalent organic frameworks were synthesized and treated with NaOH and HCl using hexamethylene diisocyanate and thiourea as amine sources for well-known isocyanate–amine reactions. 

Recently, activated carbon obtained from different organic sources such as lignin, hemicelluloses, the cellulose structure of woody plants, grasses, and agricultural residues is widely used as an adsorbent due to their non-toxic, economical, and especially high removal efficiency capabilities for organic and inorganic pollutants [31]. Numerous studies were reported to remove MB or MO from industrial wastewater using these different activated carbon materials [31]. However, activated carbons are prepared by two main steps that are the carbonization process, i.e., pyrolysis, hydrothermal carbonization, and microwave irradiation at high temperatures as 450–900 °C, and then the activation process by physical and chemical activations. Adsorbent efficiencies are mainly dependent on the preparation process and types of these processes that can increase the cost of the activated carbons [31]. COFs can be used as an alternative adsorbent because of the one-pot synthesis from low-cost precursors. The synthesized COF structures were characterized by Fourier Transform Infrared (FT-IR) spectroscopy, scanning electron microscopy (SEM), thermogravimetric analysis (TGA), zeta potential, and N_2_ adsorption/desorption measurements. After that, synthesized COF structures with adjustable surface charges and porosities were used for the removal of methylene blue (MB) and methyl orange (MO) dyes from aqueous media. In addition, various adsorption isotherms and kinetic models were applied, and the important factors for absorption, such as the partition coefficient, the selective coefficient, and the relative selectivity coefficient, were calculated.

## 2. Materials and Methods

### 2.1. Materials

Thiourea (T) and hexamethylene diisocyanate (HMDI) with high purity were supplied by Sigma-Aldrich (Burlington, MA, USA) or Merck (Rahway, NJ, USA) and used as starting materials for the synthesis of TH COF without further purification. Distilled water (DW) and acetone (99.9%, Sigma-Aldrich, USA) were used as solvent pairs in the synthesis of TH COF. Hydrochloric acid (HCl, 36.5%, Sigma-Aldrich) and sodium hydroxide (NaOH, 99.5–100.5%, Sigma-Aldrich) were used for protonation and deprotonation of TH-based COFs. Methylene blue hydrate (MB, 97%, Sigma), and methyl orange (MO, Reag. Ph. Eu., Fluka) was used as organic dyes for absorption studies.

### 2.2. Synthesis of TH-Based COFs 

Thiourea (6.5 mmol; 0.49 g) was weighed in a 100 mL round bottom flask, dissolved in 50 mL distilled water, and stirred at room temperature for 10 min until a homogeneous medium was obtained. Hexamethylene diisocyanate (6.5 mmol; 1.09 g; 1.035 mL) was dissolved in acetone (5 mL), and the solution was added dropwise to the reaction mixture for 5 min. The cloudy mixture was stirred for 20 h, eventually forming a solid white precipitate. The resulting precipitate was filtered, washed with excess water, and dried in air. The bare TH COF product was stored in a closed container at +4 °C for further use. Moreover, the synthesized TH COFs were treated with 100 mL of 2.5 M HCl and NaOH separately for 2 h for the protonation and deprotonation of TH-based COF to obtain p-TH and dp-TH COFs that will further be used for absorption studies.

### 2.3. Characterization of TH-Based COFs 

A Fourier Transform Infrared (FT-IR) spectrometer was used for the structural characterization of TH-based COFs. The analyses were performed on a Thermo, Nicolet iS10 model FT-IR spectrometer equipped with attenuated total reflectance (ATR) in a spectral range of 4000–650 cm^−1^, with 4 cm^−1^ resolution. 

The thermal behavior of TH-based COFs was followed by thermogravimetric analysis (TGA) using a TGA analyzer (TGA, SII TG/DTA 6300, Exstar) under a nitrogen atmosphere, 200 mL/min with a heating rate of 10 °C/min up to 1000 °C.

The surface charges of the prepared TH-based COFs were determined at 25 °C using a Zeta Potential Analyzer (BIC, Brookhaven Inst. Corp, Holtsville, NY, USA). For this purpose, TH-based COFs (1–10 mg/mL) were suspended in 10^−3^ M KCl solution and placed in polystyrene cuvettes. In addition, zeta potential values of TH COFs were also determined at pH 2, 4, 6, 8, 10, and 12 under the same conditions. The pH of the solutions was adjusted using 0.5 M HCl or 0.5 M NaOH solution.

BET analysis of TH-based COFs for specific surface area, pore volume, and pore size were evaluated using the Brunauer–Emmett–Teller (BET) method and Barrett–Joyner–Halenda (BJH) method with a surface area and porosity measuring device (Micromeritics, TriStar II, Atlanta, GA, USA). Prior to measurements, TH-based COFs were dried in a freeze dryer and passed through N_2_ gas for 8 h to remove moisture and other contaminants at 80 °C using a Flow Prep 060 Degasser before adsorption analysis. Subsequently, N_2_ adsorption–desorption studies in liquid nitrogen were performed to determine the pore properties of the prepared TH-based COFs.

Scanning electron microscopy (SEM, NovaNANOSEM 50 FEI) was used to obtain information about the surface topography of the prepared TH-based COFs. The SEM images were acquired after placing TH COFs on the carbon tape, attaching SEM stubs, and then coating with a few nm thicknesses of gold at 5 kV operating voltages.

### 2.4. Absorption Study 

Methylene blue (MB) and methyl orange (MO) were used as dyes for the absorption studies. Dried TH-based COFs, 50 mg were added into a 100 mL of 25 ppm dye solution and stirred at 500 rpm mixing rate. In addition, the changes in the absorbed amount of MB and MO were compared for p-TH COF and dp-TH COF structures. For this purpose, 50 mg of p-TH COF and dp-TH COF were placed into 100 mL MB and MO solutions of known concentrations (200, 100, 50, 25, and 10 ppm), respectively. A sample of 1 mL for MB and 2 mL for MO were taken from the absorption medium at specific time intervals and each sample was diluted 5 times for MB and 2 times for MO with DW. Finally, the diluted samples were measured by UV-Vis spectrophotometer at 664 nm and 464 nm to determine the absorbance of MB and MO, respectively. All the measurements were performed in triplicate and the amount of MB and MO dyes absorbed by TH-based COFs were calculated from the decreasing absorbance values at corresponding wavelengths.

### 2.5. Absorption Isotherms and Kinetics 

MB and MO absorptions by dp-TH and p-TH COFs were analyzed using five different isotherm models: Langmuir, Freundlich, Temkin, Elovich, and Dubinin–Radushkevich isotherms [32,33,34]. The details of the isotherm models are given in Supporting Information. 

The absorption studies were analyzed by applying pseudo-first-order (Equation (1)) and pseudo-second-order (Equation (2)) models in order to determine the kinetic parameters such as rate constants and absorption capacities of MB and MO from aqueous media with dp-TH COF, and p-TH COF, respectively.

log (q_e_ − q_t_) = logQ_t_ − (k_1_/2.303)t (1)

t/q_t_ = 1/k_2_q_e_^2^ + t/q_e_ (2)

where “q_e_” is the absorbed amount of molecule at equilibrium (mg/g), “q_t_” is the absorbed amount of molecule at time t (mg/g), k_1_ is the rate constant for the pseudo-first-order model (min^−1^), and k_2_ is the rate constant for the pseudo-second-order model (g.mg^−1^.min^−1^).

### 2.6. Distribution, Selectivity, and Relative Selectivity Coefficients 

The well-known and important coefficients for absorption studies were calculated for MB, and MO dyes (100 mL, 25 ppm) using bare TH, dp-TH, and p-TH COFs, by using Equations (3)–(5) [35,36]:

K_d_ = (q_e_/C_e_) . (W_adsorbent_/V_adsorbate_) (3)

where “K_d_” is the distribution coefficient, “q_e_” is the amount of adsorbate per unit weight of adsorbent (mg.g^−1^), and “C_e_” denotes equilibrium concentration of a given solute in the bulk solution (ppm) of MB or MO solution. W_adsorbent_ is the amount of adsorbent in g and V_adsorbate_ is the volume of the dye solution in L.

k = K_d_(MB)/K_d_(MO) and k = K_d_(MO)/K_d_(MB) (4)

where “k” is the selectivity coefficient, and “K_d_” is the distribution coefficient of MB or MO solution.

k^ı^ = k(dp − TH)/k(TH) and k^ı^ = k(p − TH)/k(TH) and k^ı^ = k(p − TH)/k(dp − TH) (5)

where “k^ı^” is the relative selectivity coefficient, and “k” is the selectivity coefficient of TH based COFs.

### 2.7. Reusability of dp-TH and p-TH COFs

The reusability of the prepared dp-TH and p-TH COFs for the absorption of MB and MO, respectively, was investigated by consecutive absorption–desorption studies. For this purpose, 50 mg of dp-TH COFs were placed into 100 mL 25 ppm MB and 50 mg of p-TH COFs were placed into 100 mL 25 ppm MO solution separately and stirred at a 500 rpm for 2 h for the absorption of dyes by corresponding COFs. Then the dye-absorbed COFs were centrifuged to remove the unabsorbed dye. After that, the MB-absorbed dp-TH COFs were placed into 100 mL 2.5 M NaOH, and MO-absorbed p-TH COFs were placed into 100 mL 2.5 M HCl solution and stirred at 500 rpm for approximately 1 h for the desorption of corresponding dyes. The dp-TH and p-TH COFs were then centrifuged again to collect COFs and washed once with water to remove excess NaOH and HCl. Then these COFs were placed into 100 mL 25 ppm dye solution again for the new absorption–desorption cycle. These cycles were repeated five times. The absorbed amount of MB or MO in the first cycle was considered as 100% and the amount of desorbed and subsequent absorbed amounts were compared with this value.

## 3. Results and Discussion

### 3.1. Synthesis and Characterization of TH-Based COFs

The reactions of isocyanates against nucleophilic groups under moderate conditions are reported below [37,38,39,40,41].

Reaction One.→R-NH_2_ + OCN-R’→R-NH-CO-NH-R’ (urea)

Reaction Two.→R-NCO→R-NH_2_ (amine) + CO_2_

Reaction Three.→R-NH_2_ + OCN-R’→R-NH-CO-NH-R’ (urea)

**Figure 1:**
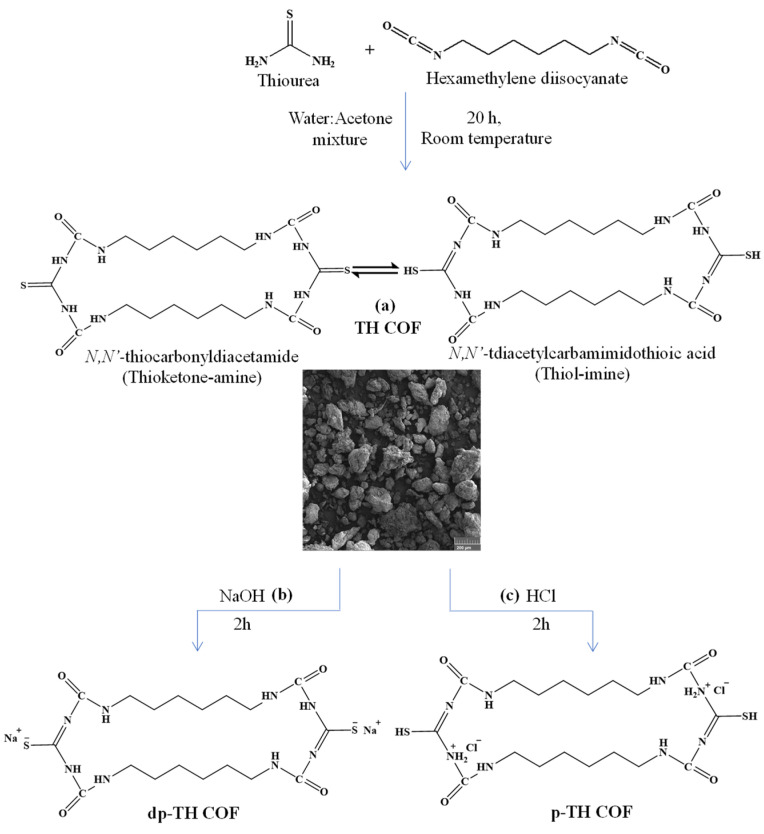
(**a**) Possible chemical structure and tautomerism of (**a**) TH COFs and the corresponding SEM image, and the change in chemical structure of TH COFs, (**b**) upon NaOH treatment (dp-TH-COF), and (**c**) upon HCl treatment (p-TH-COF).

As seen in Reaction One, urea is formed by the reaction of isocyanate with primary amines, and in Reaction Two, when it interacts with water only, carbon dioxide is released, and the amine is formed. It was also seen from Reaction Three that urea is formed again from the interaction of amine and isocyanates in an aqueous medium. It is well-known that the reaction between isocyanate and amine gives urea derivatives, while the reaction between isocyanate and urea produces biuret derivatives. Similarly, the addition polymerization reaction between diisocyanate and diamine results in polyurea as a product [42]. In this context, TH COFs were synthesized in a water/acetone solvent pair at room temperature for 20 h by the addition polymerization reaction by the reaction of thiourea (T) and hexamethylene diisocyanate (HMDI) as diamine and diisocyanate derivatives, respectively. The synthesis of TH COFs is shown schematically in Figure 1a.

As shown, in the acetone–water mixture, thiourea, which has a higher nucleophilic power will react with hexamethylene diisocyanate to form a polyurea compound. Ambient water is not expected to react with hexamethylene diisocyanate. Both amine groups in thiourea can react with both of the isocyanate groups to form polyurea compounds. In moderate conditions, a one-to-one reaction of thiourea and hexamethylene diisocyanate is more likely, and the network structure is formed. Therefore, it was shown that the smallest ring structure can be formed by the reaction of 2 moles of thiourea and 2 moles of hexamethylene diisocyanate. Otherwise, longer chain structures may occur and should be expected. The possible tautomerism was also given in Figure 1a between thioketone-amine and thiol-imine forms. The schematic presentation of the deprotonation and protonation of TH COFs are also illustrated in Figure 1b,c respectively. With the addition of a strong base to the thiol-imine tautomer, sodium thiolate is formed, and with the addition of a strong acid to the thiol-imine tautomer, thiol-ammonium chloride is formed. 

The SEM analysis was used to explore topographic information about the TH-based COFs. Considering the results, changes in particle sizes after NaOH or HCl treatment were observed to be insignificant.

The FT-IR spectra of T, HMDI, and synthesized TH COFs are shown in Figure S1a. The characteristic bands at 1600 and 1482 cm^−1^ can be attributed to C=O and the deformation vibration of the NH group on the amide structure, C=S at 1083 cm^−1^ and C-S rocking at 732 cm^−1^ of T [43,44,45], and at 2251 cm^−1^ attributed to the isocyanate group (N=C=O) of HMDI, clearly observed. The prepared TH COFs’ structures were confirmed by the FT-IR spectrum where the N-H stretching of amide groups at 3371 cm^−1^, C-H stretching peaks at 2932 and 2855 cm^−1^, and C=O and N=-H bending peaks from amide groups at 1618 and 1590 cm^−1^ can be identified. The FT-IR spectrum of TH-based COFs shows the peaks at 1076 and 732 cm^−1^ that could be attributed to C-S stretching.

The synthesized TH COFs were treated with NaOH for deprotonation of TH (dp-TH), and with HCl to protonate TH (p-TH) COFs. The TGA thermograms of bare TH, p-TH, and dp-TH COFs are illustrated in Figure S1b. There is no significant change in the thermal degradation profile of TH COFs after protonation (p-TH) or deprotonation (dp-TH) steps. Binary degradation steps were observed for TH COF with a 53.5% weight loss between 245–400 °C, and a 99.4% cumulative weight loss between 430–660 °C. Similarly, dp-TH COF also had two degradation steps between 245–380 °C and 430–630 °C with a 52.8% and 98.1% cumulative weight loss, respectively. The thermal degradation of p-TH COF was completed in three steps, which are between 220–290, 430–470, and 475–650 °C with a 44.2, 66.2, and 98.7% cumulative weight loss, respectively. It was further demonstrated that the thermal stability of TH COFs is similar to dp-TH COF with an initial degradation temperature of 245 °C, which slightly decreased after the preparation of p-TH COFs with an initial degradation temperature of 220 °C.

**Figure 2:**
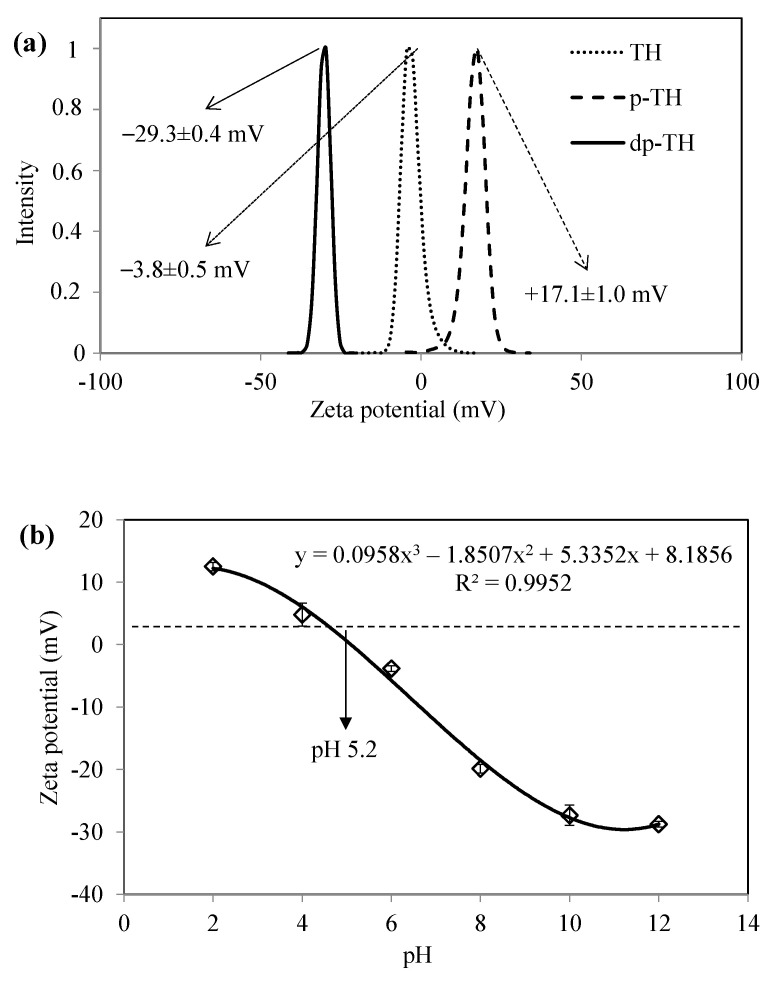
(**a**) Comparison of zeta potential of TH, dp-Th, and p-TH COFs, and (**b**) pH vs. zeta potential plots of TH COFs.

The surface charges of bare TH, dp-TH, and p-TH COFs were determined with zeta potential measurements, and the corresponding graph is given in Figure 2a. The zeta potential value of bare TH was measured as −3.8 ± 0.5 mV, whereas it was measured as −29.3 ± 0.4 mV and +17.1 ± 1.0 for dp-TH and p-TH COFs, respectively. The treatment of synthesized TH COFs with NaOH solution was observed to cause a significant reduction in the zeta potential, due to the activation of hydroxyl groups on TH COFs. Similarly, the zeta potential of TH COFs increased to approximately +17 mV when treated with HCl solution. The reason for this increase in the zeta potential of TH COFs is the protonation of amine groups. As a result of obtaining tunable surface charges of TH-based COFs, we focused on the absorption of various organic dyes from water by using TH, dp-TH, and p-TH COFs.

Figure 2b shows the zeta potential values of TH COFs measured at different pHs. The zeta potential of TH COFs was observed to decrease from pH 2 to pH 10 linearly and a small decrease was observed from pH 10 to pH 12. Moreover, the isoelectric point of TH COFs was determined as pH 5.2, approximately.

**Figure 3:**
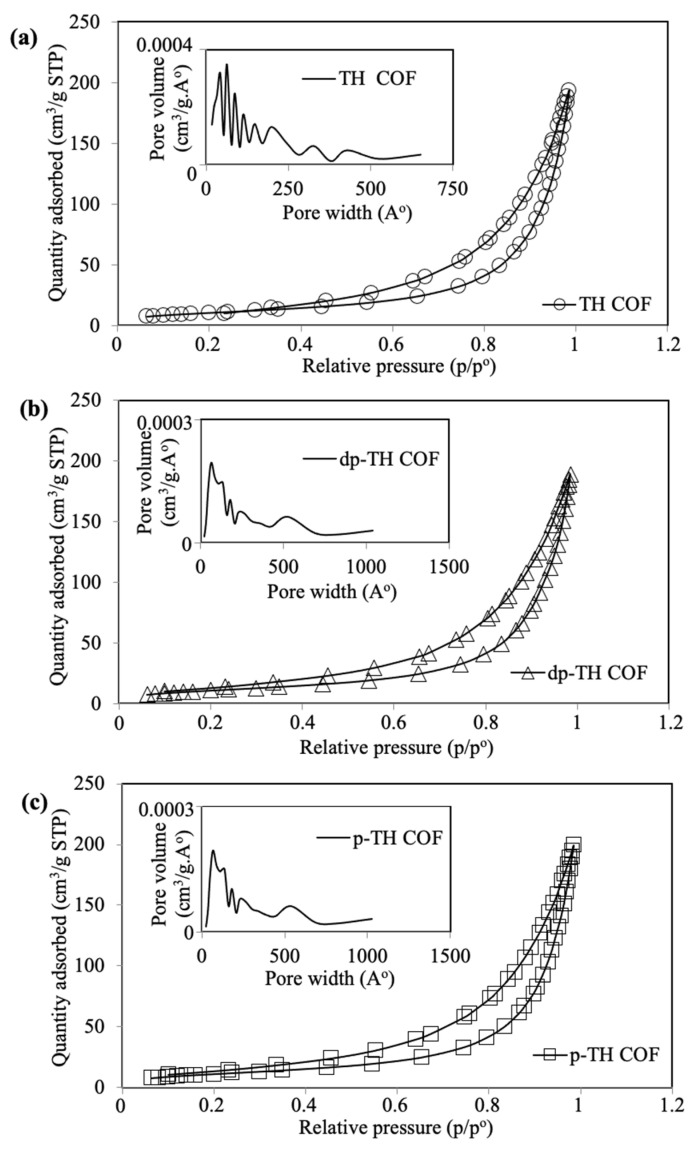
N_2_ adsorption/desorption isotherms, and pore width vs. pore volume plots (insets) of (**a**) TH, (**b**) dp-TH, and (**c**) p-TH COFs.

The surface area (m^2^/g), pore volume (cm^3^/g), and pore size (nm) values of prepared TH, dp-TH, and p-TH COFs were determined by using BET and BJH methods, and corresponding N_2_ adsorption/desorption graphs are given in Figure 3a–c, respectively. The characteristic type V isotherms and H3 hysteresis loops were observed for all TH, dp-TH, and p-TH COFs, which are attributed to the slit-like mesoporous structures of materials [46]. The pore width (A^o^) vs. pore volume (cm^3^/g.A^o^) graphs of corresponding TH-based COFs are given as insets in Figure 3. The surface area of TH COFs was 39.3 ± 1.6 m^2^/g, with 0.29 ± 0.01 cm^3^/g pore volume and 15.2 ± 3.2 nm average pore size values. On the other hand, the surface area of dp-TH COFs was measured as 41.4 ± 2.9 m^2^/g, with 0.29 ± 0.03 m^3^/g pore volume, and 15.1 ± 2.9 nm average pore sizes values. Similarly, the surface area, pore volume, and average pore size values of p-TH COFs were determined as 42.5 ± 2.1 m^2^/g, 0.31 ± 0.02 cm^3^/g, and 15.5 ± 1.9 nm, respectively. These results show that the surface area of TH COFs after NaOH and HCl treatments were almost similar as no significant differences were measured since they have almost the same morphological structures. 

### 3.2. MB and MO Absorption

**Figure 4:**
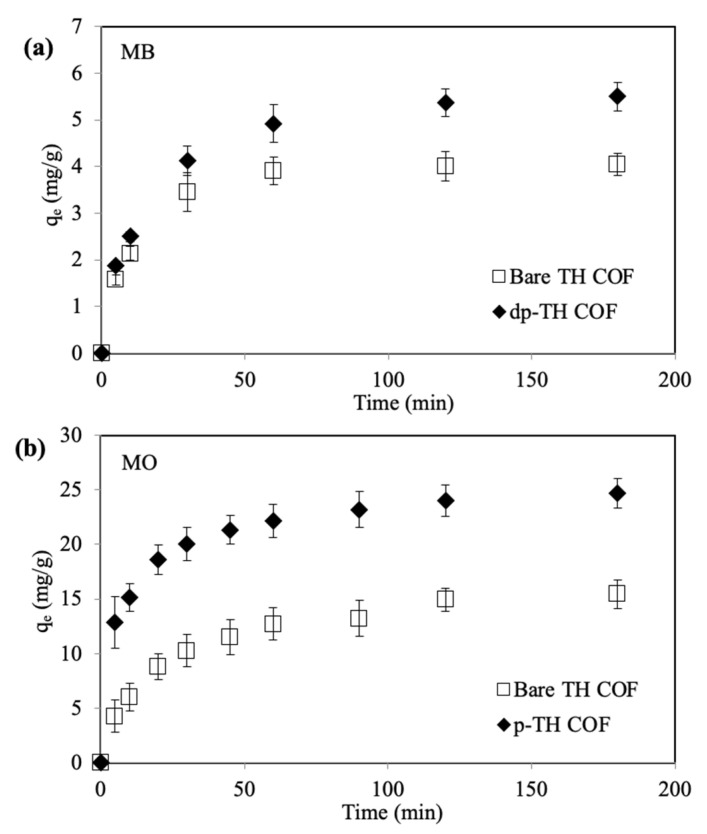
(**a**) Absorption amounts of MB dye by TH and dp-TH COFs, and (**b**) MO dyes by TH and p-TH COFs. (Reaction conditions: 100 mL 25 ppm dye solution, 0.05 g absorbent, 500 rpm, room temperature).

The absorption studies of cationic MB and anionic MO dyes were performed by using TH, dp-TH, and p-TH COFs. For this purpose, TH and dp-TH COFs were used for the absorption of cationic MB dyes, whereas TH and p-TH COFs were used for the absorption of negatively charged MO dyes. The experiments were conducted in a 25 ppm 100 mL aqueous solution of MB or MO dyes and the absorbed amounts of MB and MO dyes were calculated by the decrease in absorbance values at wavelengths of 664 nm and 464 nm, respectively. In Figure 4a, bare TH COFs absorbed a 25% lower amount of MB dye than dp-TH COFs in 120 min with a dye absorption capacity of 4.1 ± 0.2 and 5.5 ± 0.3 mg/g for TH and dp-TH COFs, respectively. The decrease in surface charges or the increase in negative charges on TH COFs after NaOH treatment provided a 25% higher amount of MB absorption from an aqueous medium. As listed in Table S1, the pH value of the MB solution was slightly increased from 6.2 ± 0.2 to 6.6 ± 0.1 in the presence of TH COF as an absorbent, on the other hand, the pH value of the MB solution was increased significantly, and was pH 8.7 ± 0.3 in the presence of the dp-TH COF absorbent in the MB solution. 

MO absorption results for the prepared TH and p-TH COFs are also given in Figure 4b. Bare TH COFs absorbed 15.4 ± 1.3 mg/g MO, whereas p-TH COFs absorbed 25.9 ± 1.4 mg/g MO in 120 min from aqueous media. The higher amount of MO absorption by p-TH COFs can be explained by the higher surface charges of p-TH COFs compared to bare TH COFs. The pH value of the MO solution in absence of the absorbent and in the presence of the TH COF absorbent was almost similar at neutral pH values, whereas the pH value of the MO solution was decreased to the acidic range, pH 3.8 ± 0.2 in the presence of p-TH COF as given in Table S1. It is clear that the pH value of the dye solutions can change to a basic or acidic range in presence of the deprotonated or protonated absorbents, however, the color and maximum absorption wavelength of MB and MO dyes were not changed in the presence of the absorbents.

**Figure 5:**
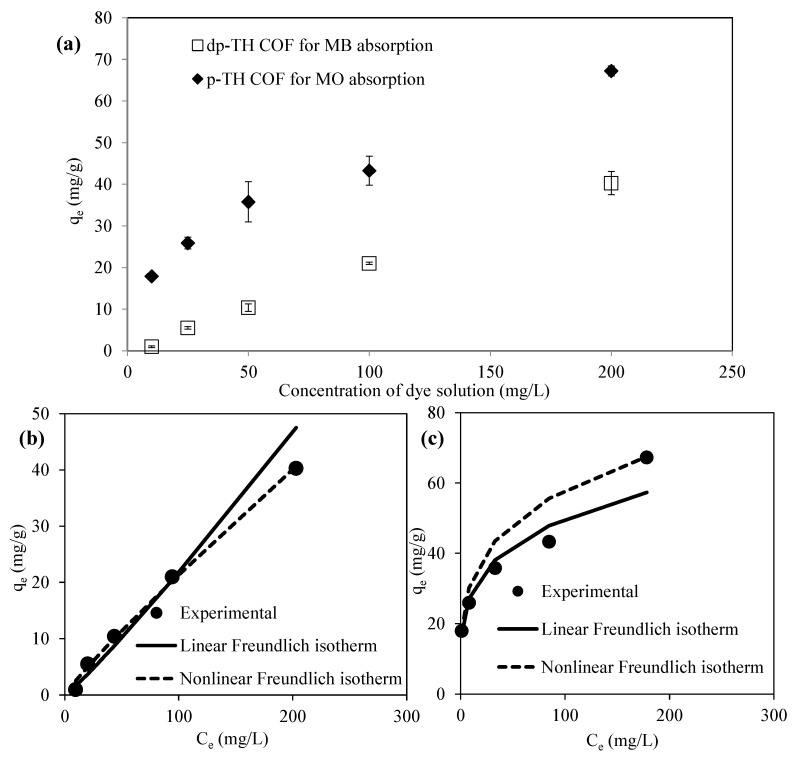
(**a**) Absorption of MB and MO dyes by dp-TH and p-TH at various concentrations of dye solutions, and curve fitting profiles of linear and nonlinear Freundlich isotherms to experimental results for (**b**) MB absorption by dp-TH, and (**c**) MO absorption by p-TH COFs. (Reaction conditions: 100 mL dye solutions at various concentrations, 0.05 g absorbent, 500 rpm, room temperature).

The absorptions of dyes from aqueous media were also investigated by varying dye concentrations, 100 mL of 10, 25, 50, 100, and 200 ppm in the presence of dp-TH COF for MB and p-TH COF for MO absorption, respectively. The corresponding graphs are presented in Figure 5a. As expected, the amount of MB absorbed from its aqueous solution by dp-TH COF increased with the increase in the MB concentration. Similarly, the amount of MO absorbed by p-TH COF increased with the increasing concentration of MO solution. Figure 5a also explains the well-known absorption isotherms, as mentioned above. 

Marsa et al. reported that apple wastes activated carbon were adsorbed at approximately 17 mg/g MO [34,47], whereas it was almost two-fold higher MO, 37 ± 3 mg/g was absorbed by p-TH COF. In another study, it was reported that 6.38 mg/g of MB was adsorbed by activated carbon prepared from waste [34,47]. These results signified that almost similar absorption capacities for both dyes can be attained by dp-TH COF. 

### 3.3. Absorption Isotherms

For the absorption process of MB and MO, five different absorption isotherms, Langmuir (linear-nonlinear), Freundlich (linear-nonlinear), Temkin, Elovich, and Dubinin–Radushkevich models, were applied by using data in Figure 5a. The plotted Langmuir, Freundlich, Temkin, Elovich, and Dubinin–Radushkevich isotherms for MB absorption are given in Figure S2a–e, and for MO absorption in Figure S3a–e, respectively. The calculated isotherm constants are summarized in Table S2. It was found that the nonlinear Freundlich isotherm can best describe the absorption of MB by dp-TH COF, and MO by p-TH COF with higher R^2^ values of 0.9981 and 0.9983, respectively. The plotted qe vs. Ce graphs with experimental, linear, and nonlinear Freundlich isotherm results are also given in Figure 5b,c, respectively. As presented in Table S2, the best fit model for absorption of MB by dp-TH COF, and MO by p-TH COF were the nonlinear Freundlich isotherm models with higher regression coefficients in comparison to the linear Freundlich model. The linear and nonlinear Freundlich model is based on the principle that surface energies are heterogeneous during the adsorption process [34,47].

### 3.4. Absorption Kinetics

The two most commonly used kinetic models, pseudo-first order and pseudo-second order models, were applied in the dye absorption studies to identify the rate and mechanism of absorption. The calculated values of qe, k_1,_ and k_2_ values from the plots of log (q_e_ − q_t_) vs. t for pseudo-first-order model, and t/q_t_ vs. t from pseudo-second-order model are given in Table 1 for both MB and MO absorptions. It was observed that the pseudo-second-order model had better fit to the data for the absorption of both MB and MO by using dp-TH COF, and p-TH COF, with 0.999 correlation coefficient (R^2^) for both absorptions. On the other hand, the calculated q_e_ values of MB absorption by dp-TH COFs were 4.2 mg/g for pseudo-first-order model and 5.9 mg/g for pseudo-second-order model, whereas the experimental q_e_ value for MB absorption by dp-TH COF was 5.5 ± 0.3 mg/g.

Moreover, the calculated q_e_ values for MO absorption by p-TH COFs were obtained as 13.3 mg/g for pseudo-first-order model, and 25.5 mg/g for pseudo-second-order model, whereas the experimental q_e_ value for MO absorption by p-TH COF was 25.9 ± 1.4 mg/g. The k_1_ and k_2_ values for MB absorption were observed as 0.03 min^−1^, and 0.01 g.mg^−1^.min respectively, whereas these values were 0.03 min^−1^, and 0.005 g.mg^−1^.min for MO, respectively. To summarize, the pseudo-first-order model can best describe the absorption behavior of MB and MO by dp-TH COF and p-TH COF, respectively, due to high correlation coefficients.

### 3.5. Determination of Absorption Coefficients

The important coefficients for absorption studies such as distribution (Kd), selectivity (k), and relative selectivity (kı) were calculated for TH, dp-TH, and p-TH COFs for the absorption of both MO and MB dyes. Dye mixtures of 100 mL 25 ppm MO/MB were prepared and 50 mg each of TH, dp-TH, p-TH COFs were placed into the dye mixture solutions separately. Corresponding coefficients are summarized in Table 2. Kd values for MB absorption were calculated as 0.09 ± 0.02, 0.13 ± 0.02, and 0.003 ± 0.0006, whereas Kd values for MO absorption were calculated as 0.50 ± 0.09, 0.11 ± 0.02, and 1.32 ± 0.15 for TH, dp-TH and p-TH COFs, respectively. An increase in Kd values was observed as a result of protonation or deprotonation processes. Thus, dye absorption is enhanced due to the superior ionic interaction of dp-TH or p-TH COFs with counter-charged dyes.

For MB absorption, k values were calculated as 0.19 ± 0.06, 1.27 ± 0.64, and 0.002 ± 0.0005 for TH, dp-TH, and p-TH COFs, respectively. As expected, the selectivity coefficient of dp-TH COFs for the MB dye is higher than TH and p-TH COFs. Furthermore, the k values for MO absorption were calculated as 5.57 ± 2.06, 0.93 ± 0.44, and 463 ± 98 for TH, dp-TH-, and p-TH COFs, respectively. This shows that the selectivity of TH COFs for MO dye decreased with deprotonation, whereas it increased with the protonation process.

Relative selectivities of the prepared TH, dp-TH, and p-TH COFs for MB and MO dyes were also evaluated. It was observed that the relative selectivity of dp-TH COFs was higher than TH COFs for MB dyes at 6.45 ± 2.25 and also less than TH COFs for MO dyes at 0.17 ± 0.05. On the other hand, the relative selectivity of p-TH COFs for MB was less than that of TH COF at 0.01 ± 0.003, whereas the relative selectivity of p-TH COFs for MO was higher than the relative selectivity of TH COF at 87.9 ± 26. Moreover, the calculated relative selectivity of p-TH COFs was also less than the relative selectivity of dp-TH COFs for MB dyes at 0.002 ± 0.0009 and also higher than dp-TH COF for MO dyes with 599 ± 385 relative selectivity value. 

As summarized in Table 2, the calculated Kd, k, and k^ı^ values show that the absorption order for MB dye is dp-TH > TH > p-TH COFs, whereas for MO dye it is p-TH > TH > dp-TH COFs. It was observed that the absorption capacity of TH COFs for MO dye is higher than for MB dye.

### 3.6. Reusabilities of dp-TH and p-TH COFs for MB and MO Absorption

**Figure 6:**
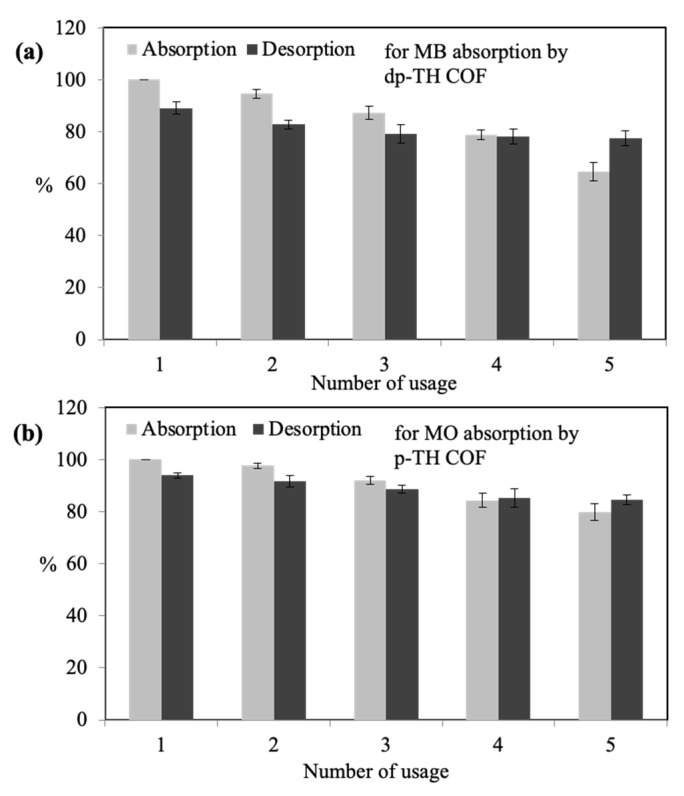
Reusability studies of (**a**) MB absorption/desorption by dp-TH COFs, and (**b**) MO absorption/desorption by dp-TH COFs. (Reaction conditions: 100 mL 25 ppm dye solutions, 0.05 g absorbent, 500 rpm, room temperature for absorption; 100 mL 2.5 M NaOH, and HCl solutions, 500 rpm, room temperature for desorption).

The reusability performance of materials is one of the most important properties for economic concerns. Therefore, the reusability of TH COFs for the absorption of MB and MO dyes were carried out in up to five absorption/desorption cycles and corresponding graphs are given in Figure 6a,b, respectively.

The absorption% and desorption% values of MB by dp-TH COFs are presented in Figure 6a. The initial absorption of dp-TH COFs was considered as 100% and the desorption value was observed as 89.1 ± 2.3% for the MB dye after NaOH treatment. After the 2nd usage, the absorption and desorption values were obtained as 94 ± 1.6% and 82.7 ± 1.8%, respectively. The absorption% of MB decreased to 87.3 ± 2.4% and 79.2 ± 3.6% of the absorbed amount after the 3rd usage. At the end of five cycles, the absorption% for MB was 64.6 ± 3.7%, whereas the desorption of MB was 77.4%.

Similarly, the absorption–desorption% values of MO by p-TH COFs are illustrated in Figure 6b. The absorption% of p-TH COFs for MO dyes decreased to 97.6 ± 1.1, 91.9 ± 1.6, 84.2 ± 2.7, and 79.7 ± 3.2% after the 2nd, 3rd, 4th, and 5th usage, where the initial absorption% of p-TH COFs for MO was considered as 100%. On the other hand, it was observed that p-TH COF desorbed 93.9 ± 1.1, 91.7 ± 2.3, 88.5 ± 1.5, 85.2 ± 3.4, and 84.5 ± 1.9% MO dye after the 1st, 2nd, 3rd, 4th, and 5th desorption processes.

It is clearly seen that dp-TH COFs preserved 64.6 ± 3.7% of absorption% ability, and also can desorb 77.4 ± 2.9% of the absorbed amount after even five usages. Furthermore, p-TH COFs maintained 79.7 ± 3.2% of the absorption% ability and also can desorb 84.5 ± 1.9% of the absorbed amount after even 5th usage.

## 4. Conclusions

In this study, an amine–isocyanate reaction was reported to prepare COF structures by using thiourea as an amine source and hexamethylene diisocyanate as an isocyanate source. It was observed that the prepared TH COF structures had tunable surface charges and surface areas with treatment by NaOH or HCl. It was demonstrated here that the protonation or deprotonation of TH COFs strongly affected the dye absorption capacity for MB and/or MO, making TH, dp-TH, and p-TH COF structures promising candidates for removing positively and negatively charged dyes from aqueous environments. Potential future studies with these types of COFs include the removal of heavy metal or other toxic organic compounds from aqueous environments.

## Figures and Tables

**Table 1:** Pseudo-first-order and pseudo-second-order constants and correlation coefficients for the absorption of MB and MO dyes by dp-TH and p-TH COFs, respectively.

Dye	Materials	Exp.	Pseudo-First-Order-Model			Pseudo-Second-Order-Model		
		Qe (mg/g)	k_1_ (1/min)	Qe (mg/g)	R^2^	k_2_ (1/min)	Qe (mg/g)	R^2^
MB	dp-TH COF	5.5 ± 0.3	0.03	4.2	0.982	0.01	5.9	0.999
MO	p-TH COF	24 ± 1	0.03	13.3	0.935	0.005	25.5	0.999

**Table 2:** Distribution, selectivity, and relative selectivity coefficients of TH, dp-TH, and p-TH COFs for the absorption of both MO and MB dyes.

Dye	Kd (TH)	Kd (dp-TH)	Kd (p-TH)
MB	0.09 ± 0.02	0.13 ± 0.02	0.003 ± 0.0006
MO	0.50 ± 0.09	0.11 ± 0.02	1.32 ± 0.15
	**k** **(TH)**	**k** **(dp-TH)**	**k** **(p-TH)**
MB	0.19 ± 0.06	1.27 ± 0.64	0.002 ± 0.0005
MO	5.57 ± 2.06	0.93 ± 0.44	463 ± 98
	**k^ı^** **(dp-TH/TH)**	**k^ı^** **(p-TH/TH)**	**k^ı^** **(p-TH/dp-TH)**
MB	6.45 ± 2.25	0.01 ± 0.003	0.002 ± 0.0009
MO	0.17 ± 0.05	87.9 ± 26	599 ± 385

## Data Availability

Not applicable.

## Supplementary Materials

The following supporting information can be downloaded at: https://www.mdpi.com/article/10.3390/mi13060938/s1, Figure S1: (a) FT-IR spectra and (b) TGA thermograms of TH based COFs; Figure S2: The (a) Langmuir, (b) Temkin, (c) Elovich, and (b) Dubinin–Radushkevich isotherms for MB absorption by dp-TH COFs; Figure S3: The (a) Langmuir, (b) Temkin, (c) Elovich, and (b) Dubinin–Radushkevich isotherms for MO absorption by p-TH COFs; Table S1: Equilibrium pH values of 25 ppm 100 mL of MB and MO solutions at the end of the absorption process by using TH, dp-TH, and p-TH COFs as absorbents and without absorbent; Table S2: Various isotherm constants for absorption of MB and MO dyes by dp-Th, and p-TH COFs, respectively [32,34,46,48,49,50].

## References

[1] Gupta V.K., Carrott P.J.M., Ribeiro Carrott M.M.L. (2009). Suhas Low-Cost Adsorbents: Growing Approach to Wastewater Treatment—A Review. Crit. Rev. Environ. Sci. Technol..

[2] Islam M.S., Tanaka M. (2004). Impacts of pollution on coastal and marine ecosystems including coastal and marine fisheries and approach for management: A review and synthesis. Mar. Pollut. Bull..

[3] Esfahani M.R., Aktij S.A., Dabaghian Z., Firouzjaei M.D., Rahimpour A., Eke J., Escobar I.C., Abolhassani M., Greenlee L.F., Esfahani A.R. (2019). Nanocomposite membranes for water separation and purification: Fabrication, modification, and applications. Sep. Purif. Technol..

[4] Babu J., Murthy Z.V.P. (2017). Treatment of textile dyes containing wastewaters with PES/PVA thin film composite nanofiltration membranes. Sep. Purif. Technol..

[5] Tan K.B., Vakili M., Horri B.A., Poh P.E., Abdullah A.Z., Salamatinia B. (2015). Adsorption of dyes by nanomaterials: Recent developments and adsorption mechanisms. Sep. Purif. Technol..

[6] Bulgariu L., Escudero L.B., Bello O.S., Iqbal M., Nisar J., Adegoke K.A., Alakhras F., Kornaros M., Anastopoulos I. (2019). The utilization of leaf-based adsorbents for dyes removal: A review. J. Mol. Liq..

[7] Reza K.M., Kurny A., Gulshan F. (2017). Parameters affecting the photocatalytic degradation of dyes using TiO2: A review. Appl. Water Sci..

[8] Karimifard S., Alavi Moghaddam M.R. (2018). Application of response surface methodology in physicochemical removal of dyes from wastewater: A critical review. Sci. Total Environ..

[9] Shooto N.D., Thabede P.M., Bhila B., Moloto H., Naidoo E.B. (2020). Lead ions and methylene blue dye removal from aqueous solution by mucuna beans (velvet beans) adsorbents. J. Environ. Chem. Eng..

[10] Azam K., Raza R., Shezad N., Shabir M., Yang W., Ahmad N., Shafiq I., Akhter P., Razzaq A., Hussain M. (2020). Development of recoverable magnetic mesoporous carbon adsorbent for removal of methyl blue and methyl orange from wastewater. J. Environ. Chem. Eng..

[11] Ghaedi A.M., Vafaei A. (2017). Applications of artificial neural networks for adsorption removal of dyes from aqueous solution: A review. Adv. Colloid Interface Sci..

[12] Wang H., Li Z., Yahyaoui S., Hanafy H., Seliem M.K., Bonilla-Petriciolet A., Luiz Dotto G., Sellaoui L., Li Q. (2021). Effective adsorption of dyes on an activated carbon prepared from carboxymethyl cellulose: Experiments, characterization and advanced modelling. Chem. Eng. J..

[13] de Salomón Y.L.O., Georgin J., Franco D.S.P., Netto M.S., Foletto E.L., Allasia D., Dotto G.L. (2021). Application of seed residues from Anadenanthera macrocarpa and Cedrela fissilis as alternative adsorbents for remarkable removal of methylene blue dye in aqueous solutions. Environ. Sci. Pollut. Res..

[14] Jarvis P., Buckingham P., Holden B., Jefferson B. (2009). Low energy ballasted flotation. Water Res..

[15] Cote A.P., Côté A.P., Benin A.I., Ockwig N.W., O’Keeffe M., Matzger A.J., Yaghi O.M. (2005). Porous, Crystalline, Covalent Organic Frameworks. Science.

[16] Ding S.-Y.Y., Wang W. (2013). Covalent organic frameworks (COFs): From design to applications. Chem. Soc. Rev..

[17] Waller P.J., Gándara F., Yaghi O.M. (2015). Chemistry of Covalent Organic Frameworks. Acc. Chem. Res..

[18] Zhu L., Zhang Y.-B.B. (2017). Crystallization of Covalent Organic Frameworks for Gas Storage Applications. Molecules.

[19] Xu H., Chen X., Gao J., Lin J., Addicoat M., Irle S., Jiang D. (2014). Catalytic covalent organic frameworks via pore surface engineering. Chem. Commun..

[20] Sahiner N., Demirci S., Sel K. (2016). Covalent organic framework based on melamine and dibromoalkanes for versatile use. J. Porous Mater..

[21] Jiang C., Tang M., Zhu S., Zhang J., Wu Y., Chen Y., Xia C., Wang C., Hu W. (2018). Constructing Universal Ionic Sieves via Alignment of Two-Dimensional Covalent Organic Frameworks (COFs). Angew. Chem..

[22] Zhan X., Chen Z., Zhang Q. (2017). Recent progress in two-dimensional COFs for energy-related applications. J. Mater. Chem. A.

[23] Sahiner N., Demirci S. (2019). The use of covalent organic frameworks as template for conductive polymer synthesis and their sensor applications. J. Porous Mater..

[24] Bai L., Phua S.Z.F., Lim W.Q., Jana A., Luo Z., Tham H.P., Zhao L., Gao Q., Zhao Y. (2016). Nanoscale covalent organic frameworks as smart carriers for drug delivery. Chem. Commun..

[25] Abdellah A.R., Abdelhamid H.N., El-Adasy A.B.A.A.M., Atalla A.A., Aly K.I. (2020). One-pot synthesis of hierarchical porous covalent organic frameworks and two-dimensional nanomaterials for selective removal of anionic dyes. J. Environ. Chem. Eng..

[26] Dinari M., Hatami M. (2019). Novel N-riched crystalline covalent organic framework as a highly porous adsorbent for effective cadmium removal. J. Environ. Chem. Eng..

[27] Wu M.X., Yang Y.W. (2017). Applications of covalent organic frameworks (COFs): From gas storage and separation to drug delivery. Chin. Chem. Lett..

[28] Zhao F., Liu H., Mathe S.D.R., Dong A., Zhang J. (2018). Covalent organic frameworks: From materials design to biomedical application. Nanomaterials.

[29] Zhao W., Xia L., Liu X. (2018). Covalent organic frameworks (COFs): Perspectives of industrialization. CrystEngComm.

[30] Tan J.C., Cheetham A.K. (2011). Mechanical properties of hybrid inorganic–organic framework materials: Establishing fundamental structure–property relationships. Chem. Soc. Rev..

[31] Kuhn P., Antonietti M., Thomas A. (2008). Porous, covalent triazine-based frameworks prepared by ionothermal synthesis. Angew. Chem.-Int. Ed..

[32] Hamdaoui O., Naffrechoux E. (2007). Modeling of adsorption isotherms of phenol and chlorophenols onto granular activated carbonPart I. Two-parameter models and equations allowing determination of thermodynamic parameters. J. Hazard. Mater..

[33] Fil B.A., Yilmaz M.T., Bayar S., Elkoca M.T. (2014). Investigation of adsorption of the dyestuff astrazon red violet 3rn (basic violet 16) on montmorillonite clay. Braz. J. Chem. Eng..

[34] Habeeb O.A., Ramesh K., Ali G.A.M., Yunus R.M., Olalere O.A. (2017). Kinetic, Isotherm and Equilibrium Study of Adsorption Capacity of Hydrogen Sulfide-Wastewater System Using Modified Eggshells. IIUM Eng. J..

[35] Dai S., Burleigh M.C., Ju Y.H., Gao H.J., Lin J.S., Pennycook S.J., Barnes C.E., Xue Z.L. (2000). Hierarchically imprinted sorbents for the separation of metal ions. J. Am. Chem. Soc..

[36] Say R., Ersöz A., Türk H., Denizli A. (2004). Selective separation and preconcentration of cyanide by a column packed with cyanide-imprinted polymeric microbeads. Sep. Purif. Technol..

[37] Vandenabeele-Trambouze O., Mion L., Garrelly L., Commeyras A. (2001). Reactivity of organic isocyanates with nucleophilic compounds: Amines; alcohols; thiols; oximes; and phenols in dilute organic solutions. Adv. Environ. Res..

[38] Vincent-Rocan J.-F., Ivanovich R.A., Clavette C., Leckett K., Bejjani J., Beauchemin A.M. (2016). Cascade reactions of nitrogen-substituted isocyanates: A new tool in heterocyclic chemistry. Chem. Sci..

[39] Bernardini J., Licursi D., Anguillesi I., Cinelli P., Coltelli M.-B., Antonetti C., Raspolli Galletti A.M., Lazzeri A. (2017). Exploitation of Arundo donax L. Hydrolysis Residue for the Green Synthesis of Flexible Polyurethane Foams. BioResources.

[40] Otálora A., Lerma T.A., Palencia M. (2019). Synthesis and characterization of polurea-based Hydrogels by Multicomponent Polycondensation of 1,6-Hexamethylenediisocyanate, sorbitol and cysteine. J. Sci. Technol. Appl..

[41] Santana J.S., Cardoso E.S., Triboni E.R., Politi M.J. (2021). Polyureas Versatile Polymers for New Academic and Technological Applications. Polymers.

[42] Sharmin E., Zafar F., Zafar F., Sharmin E. (2012). Polyurethane: An Introduction. Polyurethane.

[43] Cristiani F., Devillanova F.A., Diaz A., Verani G. (1983). Infrared vibrations of carbon—sulphur bonds in bis(benzoxazole-, benzoimidazole-benzothiazole-2-thiol)methane. Assignments by “selenation”. Spectrochim. Acta Part A Mol. Spectrosc..

[44] Tiwari D., Fermin D.J., Chaudhuri T.K., Ray A. (2015). Solution Processed Bismuth Ferrite Thin Films for All-Oxide Solar Photovoltaics. J. Phys. Chem. C.

[45] Mariappan M., Madhurambal G., Ravindran B., Mojumdar S.C. (2011). Thermal, FTIR and microhardness studies of bisthiourea-urea single crystal. J. Therm. Anal. Calorim..

[46] Buttersack C. (2019). Modeling of type IV and v sigmoidal adsorption isotherms. Phys. Chem. Chem. Phys..

[47] Halsey G.D. (1950). The role of heterogeneity in adsorption and catalysis. Discuss. Faraday Soc..

[48] Weber T.W., Chakravorti R.K. (1974). Pore and solid diffusion models for fixed‐bed adsorbers. AIChE J..

[49] Hosseini M., Mertens S.F., Ghorbani M., Arshadi M.R. (2003). Asymmetrical Schiff bases as inhibitors of mild steel corrosion in sulphuric acid media. Mater. Chem. Phys..

[50] Günay A., Arslankaya E., Tosun İ. (2007). Lead removal from aqueous solution by natural and pretreated clinoptilolite: Adsorption equilibrium and kinetics. J. Hazard. Mater..

