# Supplementary material for: Thiourea-Isocyanate-Based Covalent Organic Frameworks with Tunable Surface Charge and Surface Area for Methylene Blue and Methyl Orange Removal from Aqueous Media
Source: Micromachines (Basel). 2022 Jun 13;13(6):938. doi: 10.3390/mi13060938 (PMC9229544; doi:10.3390/mi13060938)
Supplement: Supplementary file 1 [file micromachines-13-00938-s001.zip › micromachines-1717467-supplementary.pdf]

Absoption isotherm models

According to the linear and nonlinear Langmuir model, it was assumed that the adsorption energy is uniform and there is no adsorbate migration in the surface plane [1,2], and is expressed as in Eq (1) and (2):

$$C_e/q_e = 1/q_mK_L + C_e/q_m \quad (1)$$

$$qe = q_mK_LC_e/(1 + K_LC_e) \quad (2)$$

where, "q_e" is the amount of solute adsorbed per unit weight of adsorbent at equilibrium (mg/g), "C_e" is the equilibrium concentration of the solute in bulk solution (mg/L), "q_m" is the maximum adsorption capacity (mg/g), and K_L is the Langmuir constant(L/mg).

The linear and nonlinear Freundlich models assumes heterogeneous surface energies and it becomes more heterogeneous as the value of the slope approaches zero [2-4]. Freundlich equation can be also written as Eq (3) and (4):

$$\ln q_e = \ln K_F + 1/n \ln C_e \quad (3)$$

$$qe = K_FC_e^{1/n} \quad (4)$$

where, "K_F" is a constant indicative of the relative adsorption capacity of the adsorbent ($mg^{1-(1/n)}L^{1/n}/g$) and "n" is a constant indicative of the intensity of the adsorption, and "C_e" is the equilibrium concentration of the solute in bulk solution (mg/L).

According to the Temkin model, it was assumed that the adsorption heat of all molecules in the layer decreases linearly with the coverage area due to the adsorbent-adsorbate interactions and the adsorption is characterized by a homogeneous distribution of binding energies up to a maximum binding energy [5]. Equation can be written as follows Eq. (5):

$$q_e = RT/b \ln K_T + RT/b \ln C_e \tag{5}$$

where "q_e" is the amount of solute adsorbed per unit weight of adsorbent at equilibrium (mg/g), "RT/b = B", "R" is the gas constant (8.314 J/mol K), "T" is the absolute temperature in Kelvin unit, "b" is related to the heat of adsorption (J/mol), "K_T" is the Temkin constant (L/mg), and "C_e" the equilibrium concentration of the solute in bulk solution (mg/L).

The Elovich model is based on a kinetic principle, which is assumed to increase exponentially by adsorption regions, which indicates a multi-layer adsorption [4]. The equation of Elovich isotherm is as follows Eq. (6):

$$\ln(q_e/C_e)=\ln(K_E q_m)-1/q_m \, q_e \tag{6}$$

where, "q_e" is the amount of solute adsorbed per unit weight of adsorbent at equilibrium (mg/g), "C_e" is the equilibrium concentration of the solute in bulk solution (mg/L), "q_m" is the maximum adsorption capacity (mg/g), and K_E is the Elovich constant (L/mg).

Dubinin-Radushkevich isotherm model is generally applied to express the adsorption mechanism on heterogeneous surfaces [6]. It is expressed by Eq. (7):

$$\ln q_e = \ln q_m - B\varepsilon^2 \qquad (7)$$

where "q_m" represents the adsorption capacity (mg/g), "R" is the gas constant (8.314 J/molK), and "T" refers to the temperature as Kelvin. In addition, ε^2 value is given by $\varepsilon^2=(RT\ \ln(1+1/Ce))^2$ equation.

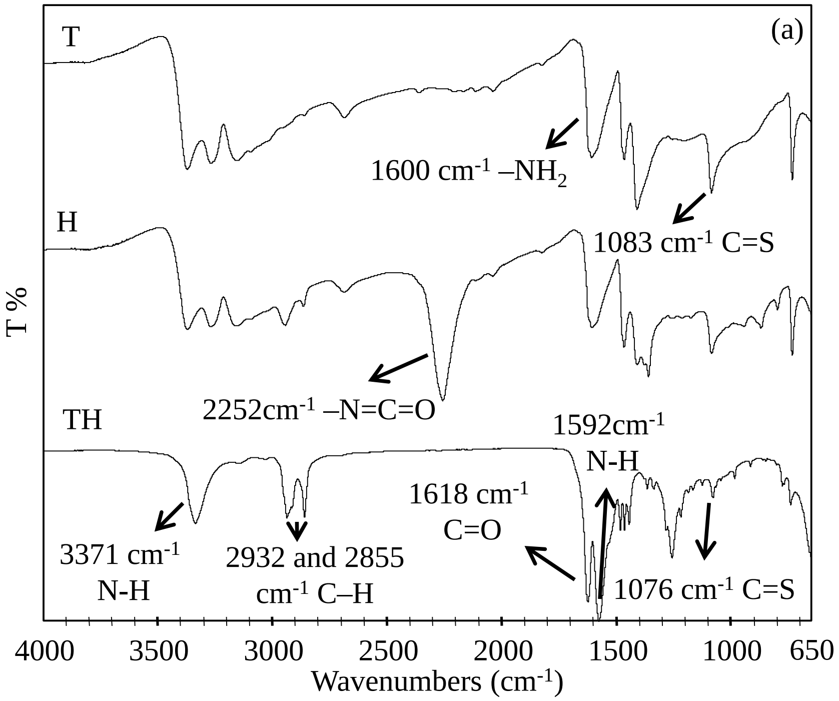

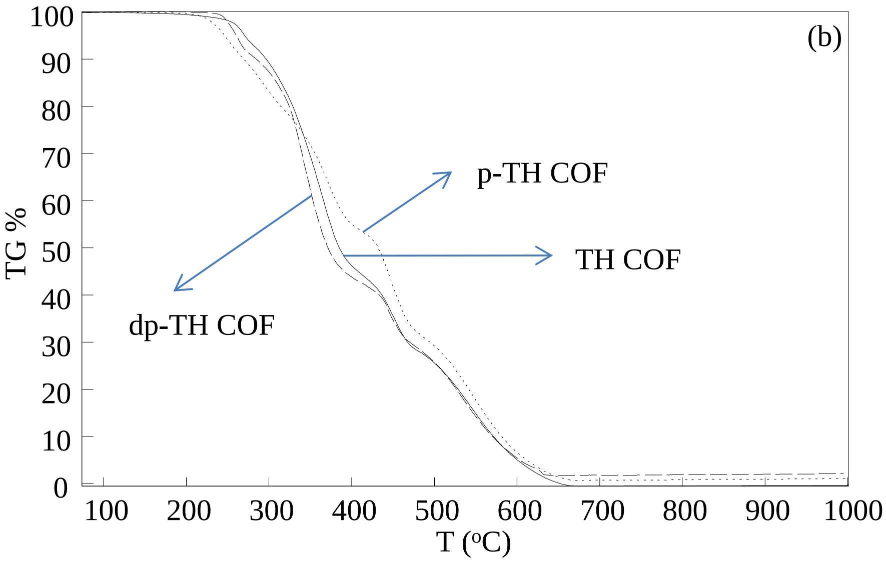

Figure S1. (a) FT-IR spectra and (b) TGA thermograms of TH based COFs.

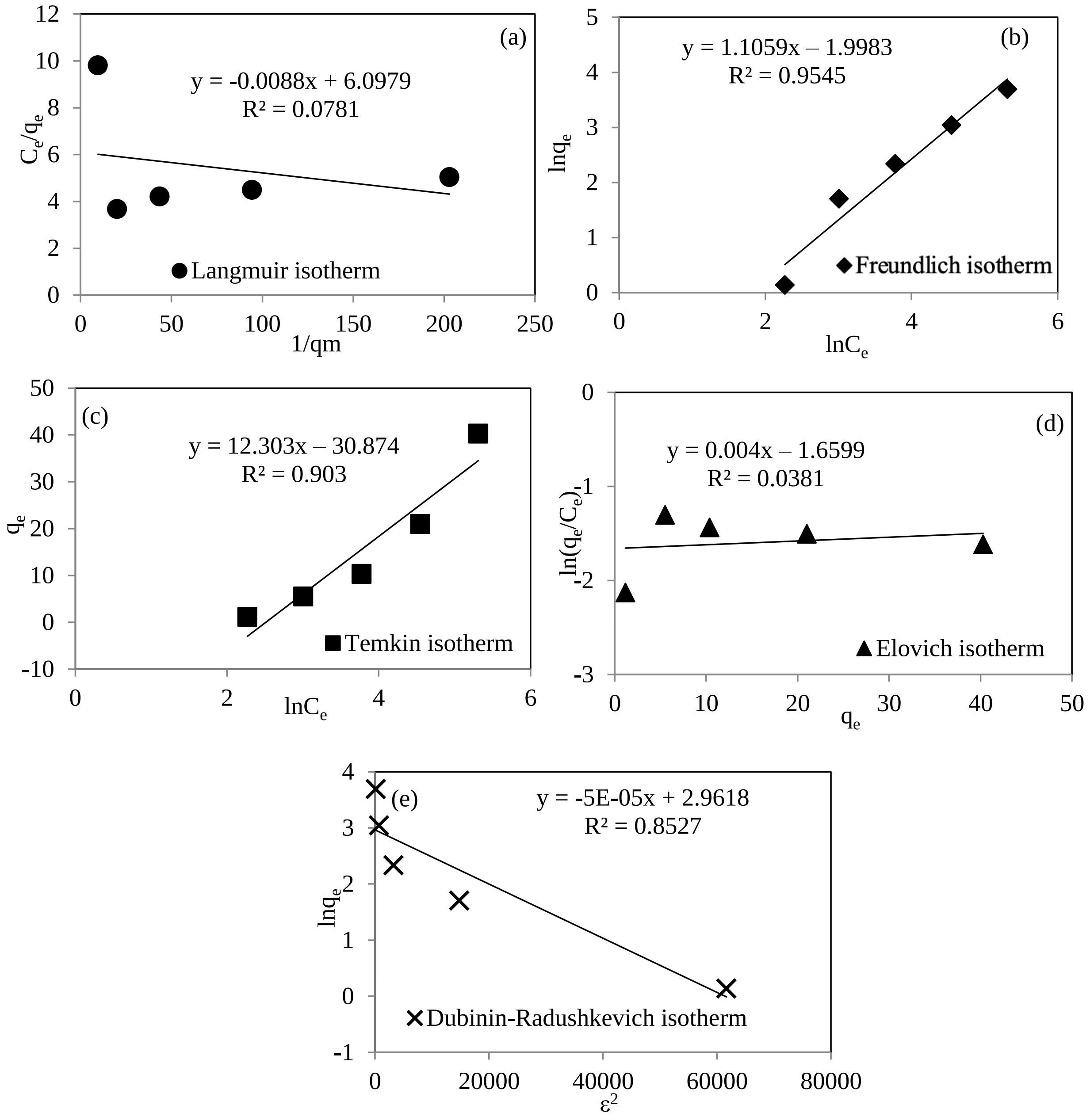

Figure S2. The (a) Langmuir, (b) Freundlich, (c) Temkin, (d) Elovich, and (e) Dubinin-Radushkecivh isotherms for MB absorption by dp-TH COFs.

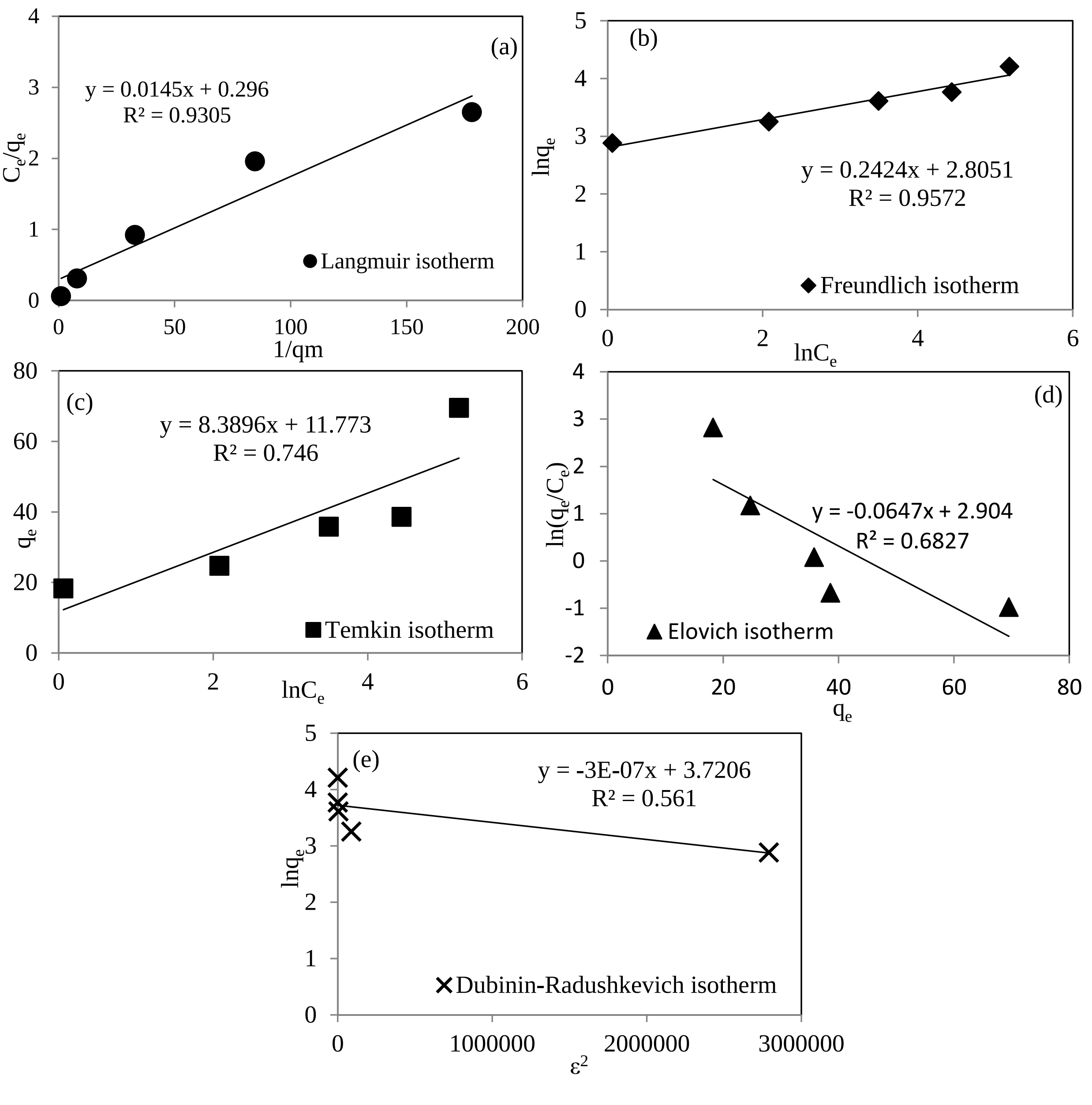

Figure S3. The (a) Langmuir, (b) Freundlich, (c) Temkin, (d) Elovich, and (e) Dubinin-Radushkecivh for MO abosrtion by p-TH COFs.

Table S1. Equilibrium pH values of 25 ppm 100 mL of MB and MO solutions at the end of absorption process by using TH, dp-TH, and p-TH COFs as absorbents and without absorbent.

**Dye**	**pH values**			
	**Without absorbent**	**TH COF**	**dp-TH COF**	**p-TH COF**
**MB**	6.2±0.3	6.6±0.1	8.7±0.3	-
**MO**	6.7±0.1	7.5±0.2	-	3.8±0.2

Table S2. Various isotherm constants for absorption of MB and MO dyes by dp-TH, and p-TH COFs, respectively.

Isotherm model	Isotherm constants				Situation
Langmuir (Linear)	Dye	**K_L (L/mg)**	**q_m (mg/g)**	**R^2**	
	MB	$1x10^{-3}$	113.6	0.078	Not fit
	MO	$2x10^{-3}$	68.9	0.930	Not fit
Langmuir (Nonlinear)	MB	$9x10^{-4}$	98.6	0.098	Not fit
	MO	$2x10^{-1}$	72.2	0.942	Not fit
Freundlich (Linear)	Dye	**K_F ($mg^{1-(1/n)}$ $L^{1/n}$ g^{-1})**	**n**	**R^2**	
	MB	$1x10^{-1}$	0.91	0.954	Not fit
	MO	16.5	4.12	0.957	Not fit
Freundlich (Nonlinear)	MB	$3x10^{-1}$	1.01	0.998	**Fit**
	MO	17.5	3.83	0.998	**Fit**
Temkin	Dye	**K_T (L/mg)**	**B (kJ mol^{-1})**	**R^2**	
	MB	$8x10^{-2}$	204.8	0.903	Not fit
	MO	4.06	300.3	0.746	Not fit
Elovich	Dye	**K_E (L/mg)**	**Q_m (mg/g)**	**R^2**	
	MB	$8x10^{-4}$	250	0.038	Not fit
	MO	1.18	15.4	0.683	Not fit
Dubinin-Radushkevich	Dye	**B (mol^2/kJ^2)**	**Q_m (mg/g)**	**R^2**	
	MB	$5x10^{-5}$	19.3	0.853	Not fit
	MO	$3x10^{-7}$	41.3	0.561	Not fit

References

1. Weber, T.W.; Chakravorti, R.K. Pore and solid diffusion models for fixed-bed adsorbers. *AIChE J.* **1974**, *20*, 228–238. https://doi.org/10.1002/aic.690200204.
2. Habeeb, O.A.; Ramesh, K.; Ali, G.A.M.; Yunus, R.M.; Olalere, O.A. Kinetic, Isotherm and Equilibrium Study of Adsorption Capacity of Hydrogen Sulfide-Wastewater System Using Modified Eggshells. *IIUM Eng. J.* **2017**, *18*, 13–25. https://doi.org/10.31436/iiumej.v18i1.689.
3. Halsey, G.D. The role of heterogeneity in adsorption and catalysis. *Discuss. Faraday Soc.* **1950**, *8*, 54. https://doi.org/10.1039/df9500800054.
4. Hamdaoui, O.; Naffrechoux, E. Modeling of adsorption isotherms of phenol and chlorophenols onto granular activated carbon. Part I. Two-parameter models and equations allowing determination of thermodynamic parameters. *J. Hazard. Mater.* **2007**, *147*, 381–394. https://doi.org/10.1016/j.jhazmat.2007.01.021.
5. Hosseini, M.; Mertens, S.F.; Ghorbani, M.; Arshadi, M.R. Asymmetrical Schiff bases as inhibitors of mild steel corrosion in sulphuric acid media. *Mater. Chem. Phys.* **2003**, *78*, 800–808. https://doi.org/10.1016/S0254-0584(02)00390-5.
6. Günay, A.; Arslankaya, E.; Tosun, İ. Lead removal from aqueous solution by natural and pretreated clinoptilolite: Adsorption equilibrium and kinetics. *J. Hazard. Mater.* **2007**, *146*, 362–371. https://doi.org/10.1016/j.jhazmat.2006.12.034.
